# Efficient Learning of Molecular Properties Using Graph Neural Networks Enhanced with Chemistry Knowledge

**DOI:** 10.1021/acsomega.5c07178

**Published:** 2025-11-06

**Authors:** Tetiana Lutchyn, Marie Mardal, Benjamin Ricaud

**Affiliations:** † Department of Physics and Technology, The Arctic University of Norway, Tromsø 9019, Norway; ‡ Department of Chemistry, The Arctic University of Norway, Tromsø 9019, Norway; § Department of Forensic Medicine, 4321 University of Copenhagen, 1172 København, Denmark

## Abstract

Graph neural networks (GNNs) have emerged as a powerful tool in predicting molecular properties based on structural data. While GNNs excel at identifying local patterns within molecules, their ability to capture global properties remains limited due to inherent structural challenges, such as oversmoothing and their expressivity. We build a simple GNN-based model that integrates chemistry knowledge that GNNs may have difficulties to learn. We show that this combination greatly enhances the accuracy compared with the pure GNN approach. It is on part due to the state of the art (SOTA) of much larger models, including large foundation models, and it even outperforms them in some cases on several benchmarks. With a simple approach, this study highlights some limitations of GNNs and the crucial benefit of giving GNN models easy access to global information about the graph in the context of applications to chemistry. We focus on regression tasks at the molecular level, on small-molecule data sets. We also investigated the possible localization of molecular substructures important for the GNN prediction using the SMILES encoding. We designed a GNN predicting molecule properties at the node level, allowing us to identify important nodes for the prediction. Additionally, the model’s architecture allows for efficient training with relatively modest computational resources, making it practical for widespread application.

## Graphical abstract

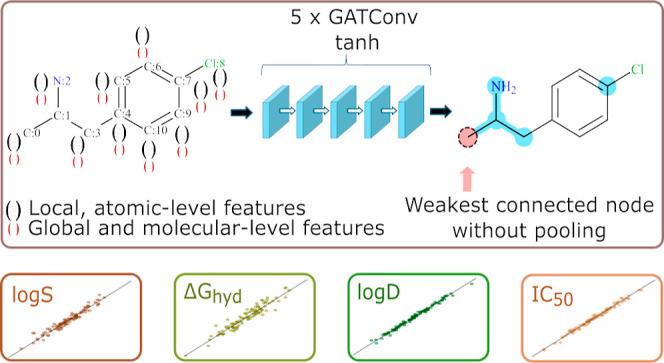

## Introduction

The development of machine learning models for chemistry is an important direction of research. Many efforts have been made in this direction in machine learning, particularly in predicting molecular properties from their structure.^1–3^ A particular kind of deep learning models, called GNNs, show extremely good results in a variety of tasks on molecular data sets.^4,5^ Indeed, the best models as of now are based on GNNs that see a molecule as a graph, with the nodes being the chemical elements and the edges being their bindings. They are able to recognize patterns inside molecules and relate them to their molecular properties.

Although GNNs are extremely good at identifying local patterns, they still struggle to identify or evaluate the more global properties of molecules. Due to their structure and their aggregation process, GNNs are prone to oversmoothing^6,7^ and have difficulties grasping information from many nodes of a graph at the same time or from nodes far apart. The expressivity of GNNs is also limited, and some simple tasks such as detecting cycles in graphs are surprisingly challenging for them.^8,9^ These limitations prevent them from identifying particular substructures or obtaining more global information about the graph geometry. It explains why approaches using expert-crafted descriptors are still competitive with GNNs in chemistry.^10^ A recent trend has been to design larger, more powerful models trained on large data sets in an unsupervised way, called foundation models. However, in many cases, these models struggle to compete with simpler machine learning methods.^11–13^

Accurate prediction of molecular properties often relies on global features on the scale of the molecule. It is essential that machine learning models have access to this information or be able to extract it from the input data. In the case of molecular geometrical properties, such as 3D shape, there exists a body of work using GNNs^14^ to predict them. So, in principle, (special types of) GNNs can get this information. However, the GNN models for these kinds of tasks are more complex and computationally heavy. For example, recent results with the Uni-Mol model,^15^ a large model based on transformers, show that 3D information is of high importance for predicting molecular properties.

In this work, we focus on regression tasks at the molecular (graph) level. We designed a simple, new machine learning model, based on a GNN, to predict molecular properties. The main goal is to demonstrate the crucial role played by global molecular properties for GNNs and their influence on the learning process of these graph-based models. While proposing a new model is not the main purpose of this work, we still give it a name for easier reference. We call it TChemGNN, for the Tiny Chemistry Graph Neural Network.

The novelty resides in two ideas. The first is to give the model easy access to the global molecular geometry. We provide global 3D features as additional input to the standard atom properties and graph. This information is derived from chemistry principles and computed from the standard molecular description. We modify the structure of the GNN to make efficient use of these additional features. The second idea is based on the hypothesis that only a substructure of the graph may contribute to the molecular property to predict. Therefore, averaging the predictions over all nodes of a molecular graph by using a pooling layer can be detrimental. We compare GNN models with and without a pooling layer. Even if a regression task at the molecular level usually necessitates a pooling layer to gather information from all nodes, here, in the “nonpooled” version, the final prediction is made by one particular node of the graph. This node (or atom) is selected by using expert knowledge from chemistry and is related to the way the molecule is encoded using the “simplified molecular-input line-entry system” (SMILES).

We show on different benchmark data sets (ESOL, FreeSolv, Lipophilicity, and BACE) that our model is as accurate and in some cases outperforms actual, much larger, models. Besides, our model is relatively small and can be trained efficiently with small computer resources. This is an important point for future applications. The code for our model is open-source and available online on Github[Fn fn1].

## Previous Work

In order to evaluate the efficiency of machine learning models on chemical tasks, several benchmark data sets have been made openly available online by the community. We choose for our regression tasks the open-source libraries ESOL,^16^ FreeSolv,^17^ Lipophilicity, and BACE.^18^ The data sets are all extracted from the Moleculenet Web site.^19^ The task of the ESOL data set is to predict the water solubility (log solubility in mol/L) for common small organic molecules, while FreeSolv provides both experimental and calculated hydration-free energy data for small molecules in water. The task for the Lipophilicity data set focuses on predicting the octanol/water distribution coefficient (logD at pH 7.4), while the BACE data set reports the binding affinity (as IC50) for a set of inhibitors of human beta-secretase 1 (BACE-1). All prediction results are evaluated using root mean squared error (RMSE), as suggested by the open repository Moleculenet.^19^ There are many publications that present new methods and test them on the data sets we have chosen. We list here the most central ones, to the best of our knowledge.

Most of the best current models are large models based on the Transformer architecture. They are trained in a self-supervised manner on large data sets. Their latent representations are then used for classification or regression tasks on other, possibly smaller, data sets. This approach is called molecular representation learning (MRL). These models are able to create their own representations of molecules and perform well in a variety of applications related to chemistry, but they are costly to train. Such models are Uni-Mol,^15^ ChemRL-GEM,^20^ and others^21,22^ for ESOL, ChemBFN^23^ for Freesolv, or ChemBERTa-2 ^24^ for BACE data sets.

Large models can be very powerful; however, we can notice a smaller architecture among the best models, called MPNN and its variants.^10^ It is based on message passing, i.e., a graph machine learning architecture. Before the MRL trend, graph neural nets were commonly (and are still) used to predict chemical properties. In this framework, the standard setting is to create a graph from a molecule with its atoms as nodes and chemical bonds as edges. Then, the atom descriptors (properties) are added as vectors of features on the nodes. The graph neural net learns to combine information from atoms and their connections for the prediction task. This approach is very effective. The study^25^ makes a systematic analysis of GNNs for small-molecule prediction tasks. They design an automated pipeline to find the best GNN architecture for a given data set. Their results are among the best for the data sets we use, showing the central importance of GNNs. As pointed out in another work,^10^ due to the small size of molecular data sets (limiting the ability to learn complex patterns) and the reduced number of message-passing layers, the focus of the GNN models is more on the local molecular structure and connections between chemical elements (local features). It is difficult to learn more global features at the scale of the entire molecule. They therefore add to their model some global molecular information at the last layer of their neural network. This is done by concatenating the latent representation with a vector of precomputed global features. In our approach, we use this idea, but we reduce the complexity by simply concatenating the global information directly at the node level and at the input. We also use a highly reduced set of global features (6 physical and 13 chemical instead of 200), with several of them related to 3D properties.

Finally, it is important to mention nondeep learning approaches that may still be competitive. Over time, chemists have developed formulas and relationships between the atomic composition of a molecule and its properties. Many of the most important and useful expert-crafted descriptors can be computed using the open-source Python library RDKIT.^26^ In particular, hundreds of molecular features can be generated from SMILES that encode the structure of a molecule. These descriptors are used in the study^10^ and in our model. In order to stress their importance, we ran a random forest regression using only these expert-crafted features and show that it gives results on par with the largest deep learning models for the FreeSolv data set.

## The Graph Neural Network Model

### Architecture

#### General Architecture

Our GNN model is shown in Figure 1. The backbone is a standard GNN made of 5 layers of Graph Attention Network “GATConv” with a hyperbolic tangent as their associated nonlinear function. The number of hidden channels in the GNN layers may vary from 32 to 64, providing similar results (the best results were obtained for 28 in all libraries). We performed a grid search to find the best hyperparameters. We also tried decreasing the embedding size through the layers (e.g., 64, 32, 16, 1), but this did not give better results. The optimizer is RMSprop. The model is relatively small, with a total number of learnable parameters of around 3.7 K.

**Figure 1:**
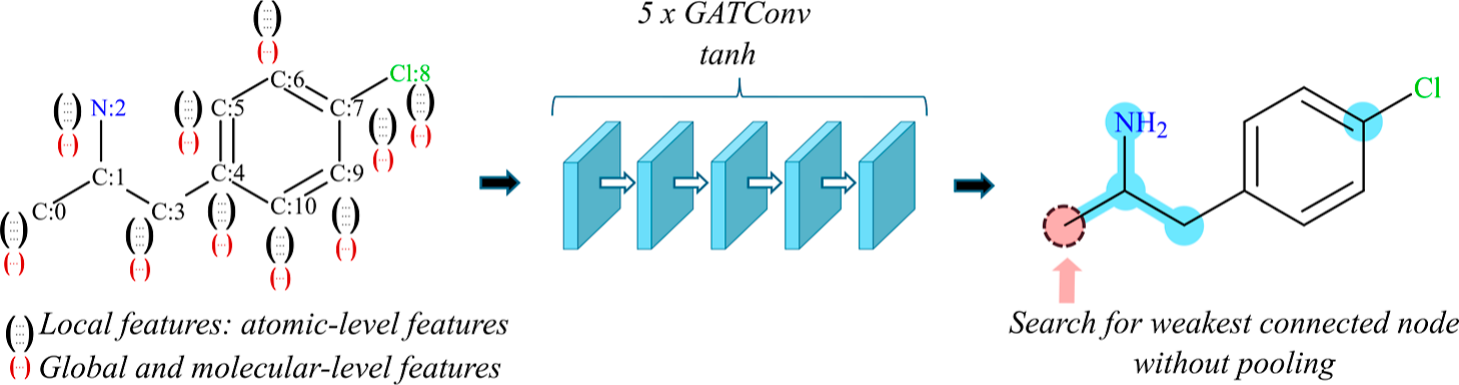
Architecture of the TChemGNN model for solubility prediction (ESOL data set). Both node features and the molecular graph are given as input to the graph neural network. The graph neural network is made of several graph attention layers (GAT), in blue. The model predicts a value per node. In the no-pooling configuration, the final predicted value is the output of one of the nodes having a particular position in the SMILES encoding. In the pooling configuration, an additional pooling layer takes the node values and performs a mean, a max, and/or a min of the node values before outputting the final prediction of the GNN.

#### No Pooling

An important new aspect of some of our models is that, at the last layer, the output is a single number given by a particular node of the molecular graph. Performing a global pooling operation (as is common with GNNs) may reduce the model expressivity^27^ and may not give the best results (neither average, min, max pooling, or the concatenation of them). Some of the molecular properties we predict may depend only on a part of the graph, and the rest provides some random noise that pooling has difficulties filtering out. To identify these key atoms, if any, we used some of the particular properties of the unique SMILES encoding. The encoding rules of the SMILES notation always put in the first position the atom with the weakest connection to the rest of the molecule.^28,29^ Following the SMILES encoding rules, the last atom in the SMILES string is also an outer atom of the molecule. Hence, by building the graph so that the node IDs correspond to their positions in the SMILES string, we are able to extract the predictions from these key positions and evaluate their contribution.

#### Input of the GNN Model

A graph neural network takes as input a graph and a matrix of features. This matrix is of size *N* × *F*, where *N* is the number of nodes (atoms in the molecule) and *F* is the number of values (features) at each node. In our case, each node of the input graph has 35 features that we keep fixed for all the data sets. A part of them are local (atom) features that describe the chemical element with which they are associated (14 features). We add to each node (representing an atom) of the graph several global features (21 features), carefully selected among the set of molecular descriptors provided by RDKIT. By concatenating these global features to the local ones, we allow direct access to important global information at the node level. We ignore the edge attributes in our model.

We detail the atomic and molecular properties below.

Atomic-Level Features (per atom): atom_degree (atomic degree (number of bonds)), atomic_number (atomic number of the atom), num_hydrogens (number of hydrogens attached to the atom), atomic_valence (atomic valence), num_radical_electrons (number of radical electrons), atom_formal_charge (formal charge on the atom), atom_hybridization (hybridization of the atom), electronegativity (electronegativity based on the atomic number), has_electronegativity (whether the atom has a defined electronegativity value), has_ implicit_hydrogens (whether the atom has implicit hydrogens), and hydroxyl_group (whether the atom is part of a hydroxyl group (−OH) based on hydrogen bonding). Additional scaled features. Note that the following last three features are scaled.^30^ The atomic mass scaled *A*_s_ is given by *A*_s_ = (*A* – 10.812)/116.092, where *A* is an atomic mass. The van der Waals radius (*R*_vdw_) of the chemical elements in a molecule is scaled as follows: *R*_vdw,s_ = (*R*_vdw_ – 1.5)/0.6. The covalent radius scaled *R*_cov,s_ is calculated according to *R*_cov,s_ = (*R*_cov_ – 0.64)/0.76, where *R*_cov_ is a covalent radius for each chemical element in a molecule.

Molecular-Level Features: has_ring (whether the molecule contains any rings), is_aromatic (whether the molecule is aromatic), formal_charge (global formal charge of the molecule), min_degree (minimum degree of atoms in the molecule), num_hbond_donors (number of hydrogen-bond donors), num_rings (number of rings in the molecule), num_rotatable_bonds (number of rotatable bonds), polar_surface_area (topological Polar Surface Area), molecular_ weight (molecular weight), num_atoms (number of atoms in the molecule), hba (number of hydrogen-bond acceptors), hbd (number of hydrogen-bond donors), fraction_sp2 (fraction of SP2-hybridized atoms), valence (global valence (sum of all atom valences)), general_electronegativity (average of all atoms’ electronegativity).

Global Features: several geometric properties of the molecule are computed using RDKIT, volume, width, length, and height of the molecule, as well as its dipole momentum and the angle of general molecular orientation. RDKIT can compute the 3D coordinates of the atoms from the SMILES. For the width (*w*), the height (*h*), and the length (*l*) of the molecule, we generated them from the 3D position of the atoms in each molecule, taking the distance between the farthest atoms in the molecule with respect to each direction, *x*, *y*, and *z*. We then combined additional RDKIT functions to obtain the dipole momentum (Merck molecular force field (MMFF) and Gasteiger partial charges) and the angle of general molecular orientation (Universal Force Field (UFF) and conformer of the molecule).

To show the importance of these spatial features, we illustrate in Figure 2 an example of 2 isomers. They have the same chemical elements but different spatial organizations with a more or less compact shape (difficult to grasp for a GNN). This difference causes changes in their behavior and properties.

**Figure 2:**
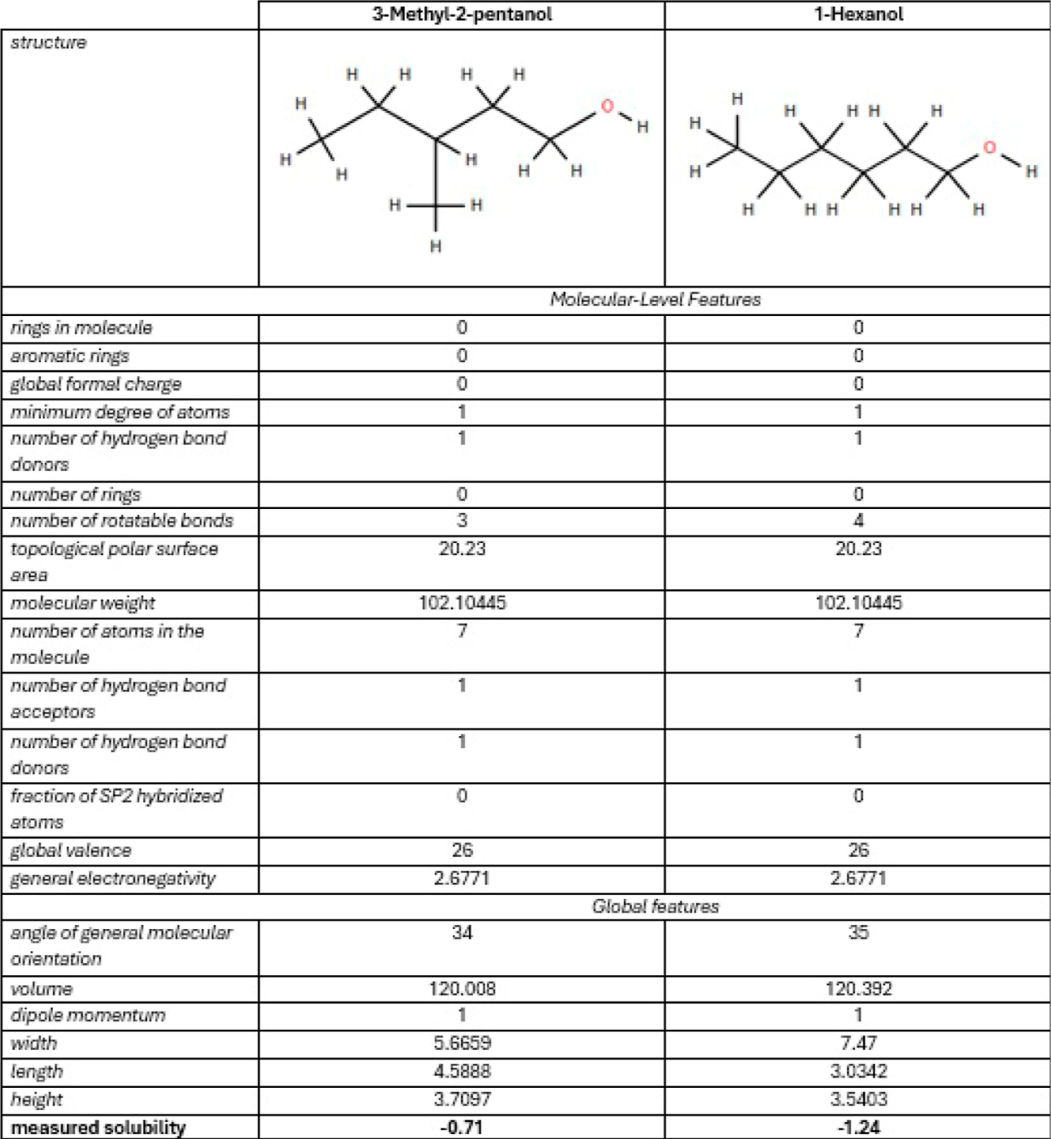
3D representations of molecules with the same chemical formula C_6_H_14_O from the ESOL library. Even the same chemical formula and a very close structure, these 2 molecules organize differently in space. The graph structure is important, but the shape in 3D as well, for predicting molecules properties. Left molecule 3-Methyl-2-pentanol and right molecule 1-Hexanol.

Unless otherwise stated, we split our data set (8:1:1) randomly, using 80% of the data for training, 10% for validation, and 10% for testing. This is the standard splitting proposed in the data repository Moleculenet.^19^

## Results

### ESOL Data Set

Our first benchmark data set is ESOL. The task is to predict the solubility of a molecule. Water solubility is given in log-scaled moles per liter. The data set contains 1128 compounds. The measured solubility values range from −8.057 to 1.071.

We list in Table 1 the best current models in this data set, to the best of our knowledge, and compare to our model.

**Table 1:** Results of Our and the SOTA Models for Solubility Predictions^a^

**SOTA**models for the ESOL library	RMSE
Uni-Mol:a universal 3D molecular representation	0.788 ± 0.029
ChemRL-GEM: geometry-enhanced molecular representation learning method (GEM) for chemical representation learning (ChemRL)	0.798 ± 0.029
SPMM: structure–property multi modal foundation model	0.817 ± 0.010
ChemBFN: Bayesian flow network framework for chemistry tasks	0.884 ± 0.003
long- and short-term memory units (LSTM)^31^	0.885 ± 0.067
ChemBERTa-2 (MTR-77M): masked-language modeling (MLM) and multitask regression (MTR)	0.889
TChemGNN (Our model)	0.7310 ± 0.0835 (0.7844)

a We display two results for our model. The first is obtained using k-fold cross-validation, using the same folds as Uni-Mol. The second, in parentheses, is the results on our test set.

In addition to this table, we can mention another work,^32^ with an even better RMSE of 0.569. However, the initial data set was reduced from 1128 to 1068 molecules. Several molecules that are difficult to classify were filtered out. While this may be relevant for chemists (gases and solids where solubility is not a meaningful property), it cannot be compared to the other ones. We focused our experiment on the full data set for the sake of clarity and a fair comparison with the SOTA. However, we performed an additional experiment, and we noticed that our results are better than the aforementioned study, on their subset (not reported here).

Our TChemGNN model provides the best results for solubility prediction and outperforms all of the models. The RMSE of our GNN model with global features is 0.7844 on the test set used to evaluate all of the models we designed. We obtain even better results from cross-validation using the same selection conditions as those used in the experiments Uni-Mol, with an RMSE of 0.7310 ± 0.0835. Uni-Mol obtain 0.788 ± 0.029.^15^

To understand better what are the key parts that lead to high accuracy in prediction, we have made an ablation study. A basic GNN model having four standard GCN layers^33^ has been implemented, the “GCN model”, to see the impact of GAT layers or other layer types. We have also modified our network, training it without global features, to evaluate their importance in the prediction. Note that the hyperparameters of the models (optimizer, step size) are different, and we selected the best results for each after having performed a grid search in the hyperparameter space. Results are reported in Table 2. The Graph attention layer and the addition of 3D features independently improve the predictions on the ESOL data set. The combination of both gave even better results. For the GNN structure, several GNN layer architectures are on par with GATs (Molecular Fingerprints MFConv,^34^ SGConv^35^) even slightly better. However, when the layer architecture and global 3D features are combined, the GAT layers gave the best results.

**Table 2:** Ablation Study for Our Model Experimented on the ESOL Library

**Basic**GCN model	RMSE
GCN model	1.0067
MFConv instead of GCN	0.9304
SGConv instead of GCN	0.8813
GATConv instead of GCN	0.8663
GATv2Conv instead of GCN	0.8930
GIN instead of GCN	1.7510
adding 3D features to GCN	0.8528
our model without 3D features	1.2351
our model with 3D features (width, length, height)	1.1884
our model with 3D features (volume, dipole momentum, angle)	1.0201
our model with 3D features (volume, width, length, height, angle)	1.1022
our model with 3D features (width, length, height, volume, polar surface area, hydrogen-bond acceptors hba, hydrogen-bond donors hbd, general electronegativity, dipole momentum, angle)	0.9507
our model with averaged results	0.7968
our model with pooling min/mean/max values	0.8756/0.7922/0.7752
our model	0.7844

In order to have a better overview of the prediction results, we show in Figure 3 the prediction error of TChemGNN for all molecules in the test set and compare it with the GCN model (on the same test set). The error does not seem to depend on the predicted value, although the solubility of the test set molecules ranges from practically insoluble to very soluble.^36^

**Figure 3:**
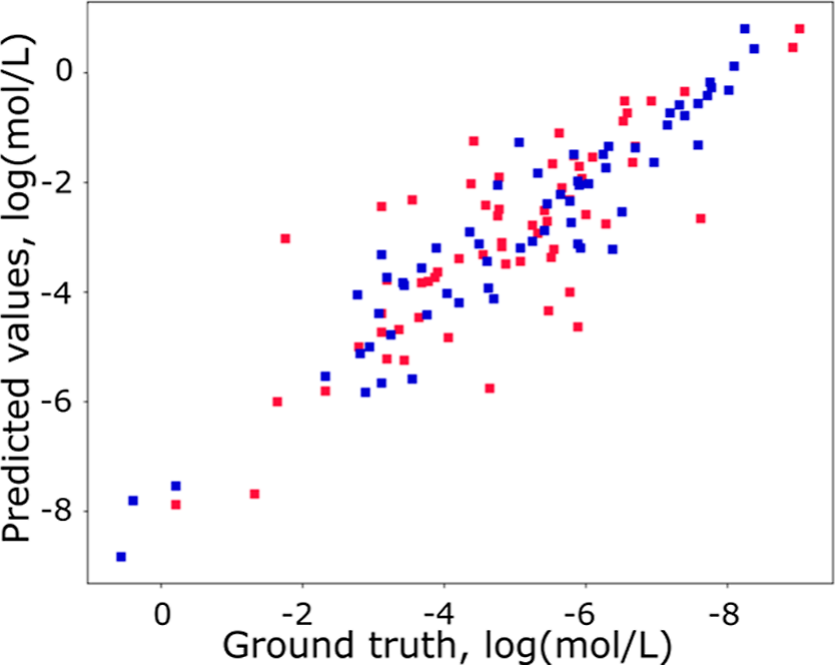
Solubility prediction with respect to the ground truth (ESOL test data set). Our model (blue dots) is compared to a basic GCN model (red). Our model is better over all the range of values.

### The FreeSolv Data Set

The FreeSolv data set is an open database with hydration free energies for a set of 642 neutral molecules, most of which are fragment-like.^19^ The data values to predict range from −25.47 to 3.43. One of the best models for this data set is ChemBFN.^23^ It is a language model trained on molecular encodings (such as SMILES). The embeddings are then used for classification and regression. It is relatively large with 54 M learnable parameters (compared to the 3.7 K of our model).

For this data set, we first performed a “simple” random forest regression using purely expert-crafted molecular features obtained from the work of Yap^37^ and RDKit. This gave an RMSE almost as good as the best deep learning model, ChemBFN. Again, expertly crafted features are extremely powerful for chemistry applications. In Table 3, we show the results of several SOTA models and compare them to our model and the random forest experiment. Our model provides the best results.

**Table 3:** RMSE of SOTA Models for Predictions of Hydration Free Energies of Small Molecules in Water

**SOTA**models for the FreeSolv library	RMSE
ChemBFN: a Bayesian flow network framework for chemistry tasks	1.418 ± 0.067
Uni-Mol: a universal 3d molecular representation learning framework	1.480 ± 0.048
GROVER (large): self-supervised graph transformer on large-scale molecular data	1.544 ± 0.072
GROVER (base): self-supervised graph transformer on large-scale molecular data	1.592 ± 0.397
SPMM: structure and properties through a single molecular foundation model	1.868 ± 0.041
ChemRL-GEM: geometry-enhanced molecular representation learning for property prediction	1.877 ± 0.071
D-MPNN: direct message-passing neural network	1.932 ± 0.412
random forest regression	1.4222
TChemGNN (Our model) validation set	1.0124
TChemGNN (Our model) cross-validation (*k* = 5)	1.1159 ± 0.1487

Considering that FreeSolv is a small data set (642 molecules), performance may be overestimated depending on the data split. To address this, *k*-fold cross-validation (*k* = 5) was applied, obtaining an RMSE of 1.1159 ± 0.1487, demonstrating the generalizability of the model.

### The Lipophilicity Data Set

The Lipophilicity data set is an open database with lipophilicity measurements from drug discovery, containing a set of 4200 small molecules. The predicted data values range from −1.5 to 4.5. We did not find any research results on this regression task. This data set was used for the GLAM study^25^ but the task was to predict the 3D structure of the molecules. We also mention Mol2vec^38^ with a RMSE = 0.589 ± 0.01 on this data set and the self-attention-based message-passing neural network (SAMPN)^39^ with RMSE = 0.571 ± 0.032. However, these studies use a particular approach that we cannot compare fairly. They train on several large libraries of molecules and modify the SMILES encoding to add more information about the structure in the input of the neural net. Moreover, they designed their network to only predict lipophilicity, while we have a more versatile network able to learn other regression tasks well.

Our model’s RMSE is 1.0222 on the test set and 1.0366 on the validation set. Note that we removed two molecules from the data set as the RDKIT function was not able to compute the dipole momentum and the angle of general molecular orientation for them.[Fn fn2]

For this task, we found that the part of the molecule that predicts the best lipophilicity is made of the nodes at the end of the SMILES encoding (Figure 4 and Table 5) and may be spread over several atoms, possibly depending on a larger molecular substructure. The mean pooling provides a slightly better prediction, showing that the single-node (atom) prediction may be too local.

**Figure 4:**
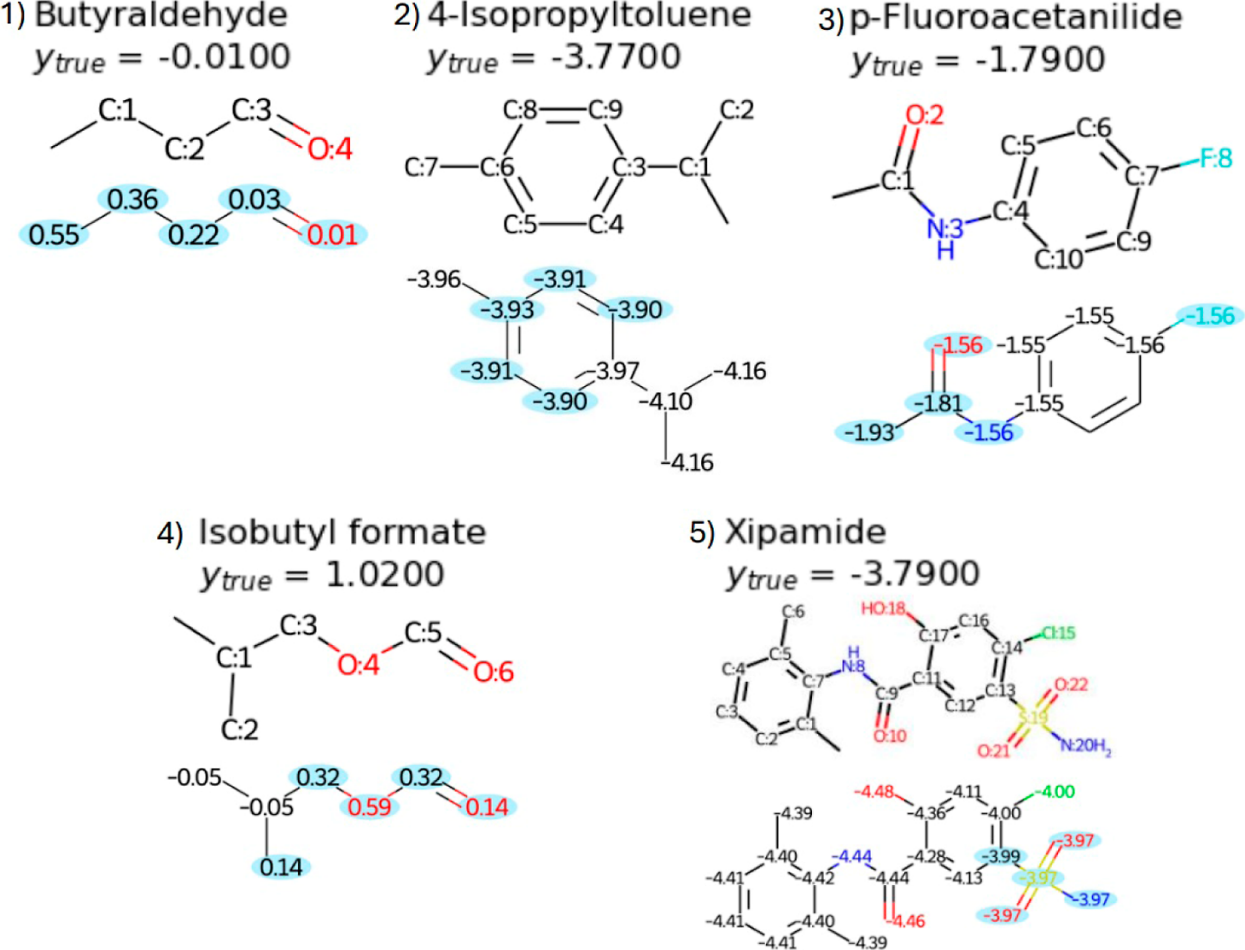
Five molecules from the ESOL library and the predictions at the atom level. For each molecule, the order of each atom in the SMILES encoding is given (top) together with the prediction at the atom level (bottom). The value *y*_true_ is the ground truth prediction for each molecule. Note: the first node in each molecule, with number 0, is omitted in the image. We selected 4 molecules where the prediction is good at the last node and one where it is not (Molecule 4 which shows a significant deviation in the solubility prediction, for all atoms).

### The BACE Data Set

The BACE data set is an open database focused on discovering drugs that inhibit the enzyme BACE, consisting of 1513 neutral molecules.^19^ The predicted data ranged from 2.5445 to 10.5229. The best model for this data set is the molecular foundation model SPMM,^22^ which is a large-scale chemical pretrained model based on 20 M SMILES representations of general molecules from PubChem.^40^ In Table 4, we present the results of the best models and compare them with those of our own. Our model provides better results.

**Table 4:** RMSE of SOTA Models for Quantitative (IC50) Results for a Set of Inhibitors of Human Beta-Secretase 1 (BACE-1)

**SOTA** models for the BACE library	RMSE
SPMM: bidirectional generation of structure and properties through a single molecular foundation model	1.041
ChemBERTa-2 (MTR-77M): toward chemical foundation models	1.363
**TChemGNN (our model)**	**0.9586**

### Results across Data Sets

We analyzed whether taking the output value at a single node or having a pooling layer (averaging or taking the max or min of the prediction over all nodes) is more suited to our tasks. We report the RMSE score at different node positions in Table 5, as well as with different pooling methods. We compare the results with randomly choosing one atom and the average prediction of 5 randomly selected ones. The best results are obtained at the last node of the SMILES or using pooling, depending on the data set. This is always better than random node selection. The results are better at the last node for ESOL and FreeSolv, using pooling for Lipophilicity and BACE. For these latter data sets, the properties may depend on more global characteristics involving a large structure inside the molecule or the entire molecule. Moreover, the results at the other nodes are much worse for ESOL and FreeSolv than it is for Lipophilicity and BACE. This is an additional argument in favor of a localized pattern (localized at the end of the SMILES) useful for the prediction on ESOL and FreeSolv, and a more global structure for Lipophilicity and BACE. It reveals the advantage of this approach for finding which part of a molecule may be influential for the prediction of a given property. In Figure 4, we show some examples of molecules with the atom order in the SMILES encoding together with the prediction at the atom level.

**Table 5:** Test Set RMSEs Are Evaluated for the Model across All Libraries, Using the Prediction at Different Positions in the Molecular Graph Corresponding to the First, Second, and Last Two Atoms in the SMILES Chain as Well as an Average of the Values from the Nodes in the Middle (All Nodes Except the First Two and Last Two)^a^

data sets/nodes	ESOL	FreeSolv	lipophilicity	BACE
1st node	2.7816	4.4491	1.3599	1.4913
2nd node	2.7733	4.4453	1.3570	1.4919
average of the nodes in the middle	0.8118	1.1423	**1**.0176	**0**.9322
one before the last node	**0**.7799	1.0444	1.0226	0.9609
last node	**0**.7844	**1**.0124	1.0222	0.9586
a single randomly selected node	0.8597	1.7299	1.0268	1.4440
average of 5 randomly selected nodes (with replacement)	0.8347	1.7481	1.0265	1.4440
average pooling	0.7922	1.8913	**1**.0174	**0**.9331

a Depending on the size of the molecule, the number of nodes in the middle varies. We removed the molecules with less than 5 atoms for this evaluation to have at least one node for the “middle position”.

Finally, the importance of adding global features to the graph nodes is shown in Table 6. The results are much better with global features for all data sets.

**Table 6:** RMSE of Our Models for the Different Libraries on Their Respective Test Sets, Using Our Best Model with and without Global Features

Libraries	without global features	with global features
ESOL	1.2351	0.7844
FreeSolv	1.3175	1.0124
Lipophilicity	1.2543	1.0222
BACE	1.4255	0.9586

## Discussion

From the literature, we know that graph neural networks perform well on molecular data. That is why we chose a GNN as a base for our model. However, GNNs have some limitations that are highlighted by our results. To overcome these limitations, we have tested several model architectures and proposed solutions combining the learning from local and global properties. Global graph properties, such as spatial geometry, are challenging to learn for GNNs. So, giving the model access to this information helps it. Rather than using an overly complicated model with the risk of overfitting, molecular features are selected for the task at hand. Several of our results show evidence of the crucial role of expertly crafted features, particularly global 3D features. First, the ablation study of the models trained on the ESOL data set shows a big difference in performance when trained with and without global features. Second, for the FreeSolv data set, a random forest run purely on chemist’s features has almost the same results as the best deep network with millions of learnable parameters. These expert-crafted features are underestimated in the machine learning literature. Enriched with global information about the molecule, our small, customized GNN model (∼3.7 K learnable parameters) has excellent accuracy and can outperform much larger models.

We propose the first approach that looks at using SMILES encoding to identify atoms important for predicting particular global molecule properties. We show that the position of an atom in the SMILES encoding can be leveraged to identify a molecular substructure that is important for the predictions. In our 4 data sets, we show evidence that the atoms in the last part of the SMILES give better predictions (Table 5). We found that it is the node corresponding to the last atom of the SMILES encoding for the regression task on ESOL, FreeSolv, and, to a lesser extent, Lipophilicity. Indeed, as discussed earlier, these extremity nodes are also, by construction of the SMILES encoding, the most peripherical nodes, with a weaker connection to the rest of the molecule. These atoms are more prone to interact with other molecules and shape-important molecular properties. Our approach aligns with basic (bio)­chemical intuition: different parameters for molecular representation affect tasks for predicting molecular properties differently. Where functional groups located in the periphery of a molecule are very important for solubility, the molecular configuration may be more important for enzyme inhibition.

In conclusion, our model performs better, thanks to the use of efficient inductive bias. We leverage knowledge from chemistry both in the input (global and 3D features) and in the structure of the neural net (choice of single-node output).

The identification of particular atoms where predictions are the best is also a way toward better explainability or interpretability in machine learning applied to molecules. Finding the atom or group of atoms in molecules where the prediction is the best gives important insights into chemists about which part of the molecule influences a property. Our approach is one new step in this direction, and it may be beneficial to connect it to the literature about explainability in GNNs in the future.

## Conclusion

GNN models are very powerful for chemistry applications but still have strong limitations. This is due to the known problems of oversmoothing and oversquashing coupled with limits in their expressivity. This prevents the models from easily grasping the global geometrical structure even for small molecules. Our study demonstrates significant improvements in predicting molecular properties when adding information about the molecular structure such as its 3D shape. We also shed light on the potential of node-level prediction for molecular-level regression tasks leveraging SMILES encoding. Further work in this direction may contribute to a better explainability of GNN predictions, with the localization of substructures of molecules important for the prediction.

In this work, we propose solutions using expert-crafted features and expertise coming from chemistry. This is valuable both from the point of view of chemistry and from that of machine learning. These solutions make the model simple, small, and efficient and hence very convenient for use in chemistry. At the same time, it highlights in a simple way the limitations that actual machine learning approaches have to overcome to improve models for chemistry and beyond.

## Data Availability

In this work, we used open-access data from Moleculenet.org.^19^ The code for our model and experiments is open-source and available at https://github.com/uitml/TChemGNN.

## Footnotes

a Code available at https://github.com/uitml/TChemGNN.

b The RDKIT function *AllChem.ComputeMolVolume* failed for the molecules in the Lipophilicity library CHEMBL387675 and CHEMBL2070121. This function is required to compute the dipole momentum and the angle of general molecular orientation.
